# Foodborne Microbial Communities as Potential Reservoirs of Antimicrobial Resistance Genes for Pathogens: A Critical Review of the Recent Literature

**DOI:** 10.3390/microorganisms11071696

**Published:** 2023-06-29

**Authors:** Paola Zinno, Giuditta Perozzi, Chiara Devirgiliis

**Affiliations:** 1 Institute for the Animal Production System in the Mediterranean Environment (ISPAAM), National Research Council, Piazzale Enrico Fermi 1, 80055 Portici, Italy; paola.zinno@cnr.it; 2 Research Centre for Food and Nutrition, CREA (Consiglio per la ricerca in agricoltura e l’analisi dell’economia agraria), Via Ardeatina 546, 00178 Rome, Italy; giuditta.perozzi@gmail.com

**Keywords:** antibiotic resistance, one health, lactic acid bacteria, conjugation, mobile elements

## Abstract

Antimicrobial resistance (AMR) is a global and increasing threat to human health. Several genetic determinants of AMR are found in environmental reservoirs, including bacteria naturally associated with widely consumed fermented foods. Through the food chain, these bacteria can reach the gut, where horizontal gene transfer (HGT) can occur within the complex and populated microbial environment. Numerous studies on this topic have been published over the past decades, but a conclusive picture of the potential impact of the non-pathogenic foodborne microbial reservoir on the spread of AMR to human pathogens has not yet emerged. This review critically evaluates a comprehensive list of recent experimental studies reporting the isolation of AMR bacteria associated with fermented foods, focusing on those reporting HGT events, which represent the main driver of AMR spread within and between different bacterial communities. Overall, our analysis points to the methodological heterogeneity as a major weakness impairing determination or a causal relation between the presence of AMR determinants within the foodborne microbial reservoir and their transmission to human pathogens. The aim is therefore to highlight the main gaps and needs to better standardize future studies addressing the potential role of non-pathogenic bacteria in the spread of AMR.

## 1. Antimicrobial Resistance (AMR) within Environmental Niches

A wealth of literature describing the spread of AMR in the environment has been published in the past few decades, with an abrupt increase since the mid-nineties and presently reaching the level of almost 15,000 papers/year. The keyword “antimicrobial resistance” retrieves a total number of publications rising from 2534 in 1995 to 13,878 in 2022, with very similar numbers when using the keyword “antibiotic resistance”. This publication trend reflects not only the growing emergence of antibiotic-resistant bacterial pathogens reported in clinical settings but also the increasing knowledge of the presence of genetic determinants of AMR in microbial reservoirs related to different environmental niches, which include a broad range of uncharacterized, non-pathogenic bacterial strains that could potentially spread AMR determinants to pathogens [1,2]. Among the environmental reservoirs of AMR, the foodborne niche raises special concern for human health, as a significant number of bacteria can reach the human gut through the food chain, where horizontal gene transfer (HGT) is more likely to occur within the crowded microbial environment of the resident microbiota. Although the foodborne reservoir mostly consists of naturally occurring, non-pathogenic strains that are commonly found in fermented foods, such as lactic acid bacteria (LAB), the possible presence of foodborne pathogens such as *Salmonella*, *Listeria*, and *Escherichia coli* as incidental food contaminants cannot be excluded [3,4]. Moreover, the resident gut microbiota is known to host a small fraction of pathogenic species. Within this context, potential transmission of AMR determinants from foodborne bacteria to human pathogens cannot be ruled out, although a causal relationship has not yet been proven.

The issue of HGT to pathogens is prejudicial for proper assessment of the risk of AMR spread from non-pathogenic environmental reservoirs, and an increasing number of recent studies have been addressing the question experimentally. However, despite two decades of research and the availability of increasingly complex molecular approaches, a clear picture of the role of environmental reservoirs in AMR transmission to pathogenic bacteria has not yet emerged (reviewed in [1]). In the present review, we will focus on the foodborne microbial reservoir through the critical evaluation of a number of recent publications reporting the isolation of AMR bacteria from foods, with the aim of showcasing the major gaps and the needs that should be considered to better standardize future studies addressing the potential role of non-pathogenic bacteria in AMR transmission to human pathogens.

## 2. The Foodborne Reservoir of AMR

Among foodstuffs, fermented products represent a very important source of live bacteria for humans. They are a complex category of foods, recently defined by an expert panel of the International Scientific Association for Probiotics and Prebiotics (ISAPP) as “foods made through desired microbial growth and enzymatic conversions of food components”, including all foods and beverages obtained through fermentation, irrespective of the presence of living microbes within the food matrix at the time of consumption [5]. The consumption frequency of fermented foods is difficult to estimate with accuracy, but they are traditionally present in all countries worldwide as they represent an ancient and effective way of preserving foods, the majority of which contain fermentable substrates. The prevalence of different fermentable food matrices varies among countries, with dairy products prevailing in Europe and South America, while plant-based foods are mostly consumed in South Asia and Africa [6]. Among foods, dairy products represent one of the principal sources of foodborne microbes ingested upon consumption due to their high content of live bacteria and yeast. The autochthonous microbiota associated with traditional fermented foods is a consortium of microbial strains of environmental origin characterized by high biodiversity [7], which reflects into a broad spectrum of metabolic pathways leading to the production of mostly volatile compounds conferring unique sensorial and organoleptic features to the final products [8,9]. Food fermentation can also be performed using commercial starters containing a few selected and well-characterized collection strains, but the great majority of food producers in all countries employ traditional production protocols that exploit the distinctive features of uncharacterized microbial consortia pre-existing in the raw materials and restricted to specific geographical environments to confer uniqueness to their products. Although the fermented food microbiota is not highly complex at the species level, its taxonomic biodiversity stems from the presence of this broad panel of environmental strains, enriching the gene pool of the microbial community (namely, the metagenome) with desirable traits of technological value. However, they could also introduce undesirable and potentially transmissible determinants, such as those responsible for AMR. Metagenomics, including metabarcoding and shotgun sequencing, are therefore promising approaches to uncovering the functionality of food metagenomes, helping to characterize the whole microbial community within a single food product, both in terms of quality and safety [8,10,11].

## 3. Horizontal Gene Transfer (HGT)

AMR can be classified into intrinsic, acquired, and adaptive. The acquisition of AMR determinants usually occurs through HGT, which represents the major mechanism through which bacteria can acquire foreign genes from the environment or from other microorganisms. It is commonly mediated by mobile genetic elements such as transposons, integrative-conjugative elements, integrons, or plasmids. In addition to these mobile genetic elements, the presence of insertion sequences (IS) within bacterial genomes is also predictive of HGT. IS are simple mobile elements capable of autonomous transposition and are often identified in association with AMR genes. IS-encoded transposases promote the formation of circular elements as transient replication intermediates, which can either integrate at different chromosomal locations or be horizontally transferred to other cells [12]. Transformation, transduction, and conjugation are the principal mechanisms of HGT, and plasmid-mediated conjugation is considered one of the main pathways for the dissemination of antibiotic resistance genes. During conjugation, the transfer of resistance genes occurs laterally, by cell-to-cell contact via pili or adhesion molecules [13]. Extracellular vesicles (EVs) represent another effective player in the exchange of bacterial AMR genes, both at the inter-species and intra-species levels [14,15]. Moreover, HGT is responsible for spreading AMR within biofilm-forming bacterial communities [16]. The most common AMR mechanisms include reduction in antibiotic intracellular accumulation through regulation of efflux pumps and decreased membrane permeability, antibiotic modification and/or inactivation within the cell, modification of the cellular targets leading to reduced sensitivity, and intracellular sequestration [17]. Within this context, and despite the huge research efforts of the past few decades (reviewed by [1]), a few important questions still remain unanswered, mainly concerning the effective transferability of AMR determinants from non-pathogenic to pathogenic bacteria and the resulting assessment of the actual risk posed by environmental bacterial reservoirs of AMR resistance for human health. The reverse transfer of AMR from pathogens to commensal bacteria as well as to the non-pathogenic components of the microflora during food fermentation has also been hypothesized to occur through HGT events (conjugation and/or phage-mediated transduction), thus enriching the AMR gene pool within environmental niches [18,19]. However, although horizontal transfer events are predicted to occur among all members of any given microbial environment, the presence of pathogens within the fermenting microbiota is generally negligible as they only occur as unwanted contaminants, and this renders the reverse flow of AMR very unlikely. To the best of our knowledge, no experimental evidence has ever been reported of the acquisition of AMR determinants from pathogens by food-fermenting bacteria. These open questions are especially relevant in the case of live foodborne bacteria, which can act as a direct link between the environment and the human body. We have recently performed a systematic review of the studies investigating the connection between foodborne and gut microbiomes, highlighting a complex interplay between the two microbial environments through the food chain. Our results confirm the suggestion that a relevant fraction of foodborne microbes is indeed able to reach the lower gut and can transiently merge with the resident microbiota [20].

## 4. AMR Detection within the Foodborne Bacterial Reservoir

As a follow-up of our previous studies and with the aim of highlighting the strengths and weaknesses that should be taken into consideration in future studies addressing the link between the two milieus, we sought to analyze in this review a comprehensive list of experimental studies published in the past 5 years reporting the isolation of AMR bacteria associated with fermented foods. The studies were retrieved by browsing the PubMed database using the search string [((antibiotic resistance) AND (lactic acid bacteria)) AND (fermented food)], and limiting the time span to articles published between 2018 and 2023. This initial search retrieved 120 research articles, but we restricted the final list to 49 articles following an accurate reading of the text, as they appeared to be the most relevant for the aims of the present review. We then focused in particular on those reporting HGT events, which represent the main driver of AMR spread within and between different bacterial communities.

**Figure 1:**
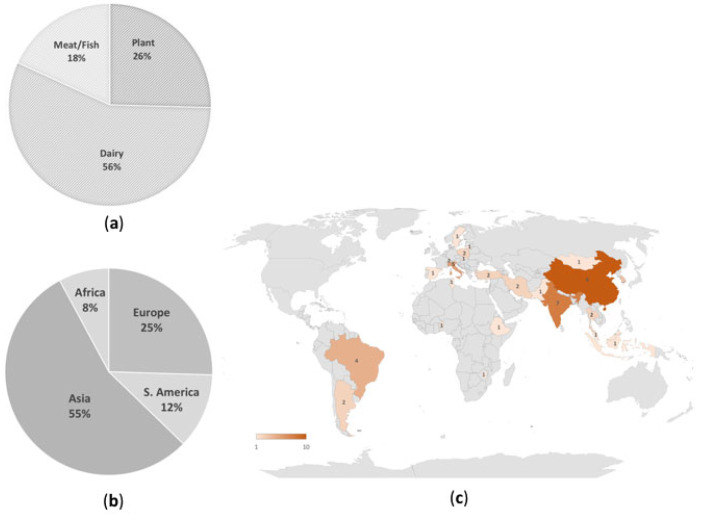
Frequency of occurrence of fermented food matrices and their geographical origin in the analyzed studies. (**a**) Distribution of the analyzed fermented foods within the three categories of food matrices (dairy, plant, meat/fish); (**b**) Geographical origin of the fermented foods, analyzed in the studies, expressed as their percentage originating from each continent. The food products originating from Turkey, a trans-continental country belonging to both Europe and Asia, were assigned to the Asian continent, which includes the vast majority of the Turkish territory; (**c**) World map showing the distribution of fermented foods analyzed in each country. The number of food products/country spans between 1 and 10 and is represented by a color gradient (the world map was created with Microsoft Excel using Bing technology).

The fermented food matrices analyzed in the articles, along with their geographical origin, are reported in Figure 1.

Overall, the great majority of studies reported the identification of AMR bacteria in animal-based fermented food products, including both dairy and meat/fish sources (Figure 1a). However, in line with the worldwide prevalence of fermented foods and beverages of dairy origin, this latter category of foods represented the great majority (76%) of the animal-based foods analyzed in the studies and collectively 56% of the total fermented foods analyzed, while other animal food sources (meat and seafood) were the least represented, accounting for 18% of the total. Vegetable, cereal, and legume-based fermented products collectively accounted for 26% (Figure 1a).

Figure 1b reports the geographical origin of the fermented food products analyzed in the present review. As shown in this panel, slightly more than half of the studies were conducted on foods originating from Asian countries (mostly China and India), where fermented foods are traditionally used to increase the nutritional value of the available raw materials (Anal 2019), followed by European countries (Italy, Lithuania, Poland, Slovakia, Spain, Sweden, and Switzerland), which contributed one fourth of the food products. Foods derived from African or South American countries were also reported, but in a more limited number of articles (8% and 12%, respectively). A detailed geographical origin of the foods produced in each country is superimposed on the world map in Figure 1c.

When compared to the geographical distribution of the studies (Table 1), the prevalence of dairy fermented foods applied to all continents with the exception of Asian and African countries, where the number of studies on dairies was equivalent to those analyzing AMR bacteria in plant-based foods. This pattern most likely reflects food habits, as plant-based foods are more frequently consumed in Asia and Africa than on other continents [6].

Table 1 summarizes the main features of the 49 articles included in the analysis, which were listed according to the country of origin of the analyzed foods (Asia, Africa, South America, and Europe).

As shown in the Table 1, we detected a broad heterogeneity among the retrieved articles in terms of experimental approaches and taxonomical analysis, as well as in the extent of molecular characterization of the AMR determinants. Such heterogeneity mostly reflects the main objectives of each study, some of which were not specifically targeted at the identification of AMR bacteria but rather pointed at a comprehensive description of the microbiota associated with fermented foods, including but not necessarily focusing on AMR. Screening of AMR strains/isolates was performed on antibiotic-containing media in only two of the retrieved studies [47,60], while in most of the others the presence of AMR determinants was identified using molecular tools (PCR, WGS, Southern blotting), and in four studies it was not confirmed by testing phenotypic resistance as well (Table 1). However, 19 studies analyzed AMR strains from existing collections that might have been originally screened on antibiotics [32,33,36,40,42,43,45,48,51,53,54,58,59,61,62,64,65,68,69]. Moreover, some of the studies focused on only one or a few specific species of technological relevance within the food microbiota without providing a comprehensive taxonomical analysis of all AMR genera/species. The number of antibiotics analyzed in each article was also very heterogeneous, spanning from one or a few to a complex panel representing several pharmacological classes.

The power of molecular approaches is increasingly being exploited to investigate AMR in foodborne bacteria, but the identification of AMR determinants by genome sequencing alone is not sufficient to assign the AMR phenotype to a specific strain or isolate, as it does not provide information on the actual expression of the detected gene(s). As mentioned above, four of the analyzed articles did not test phenotypic resistance to the antibiotics corresponding to the detected AMR determinants, whose presence was identified exclusively by sequence comparison. On the other hand, 21 of the articles reporting phenotypic resistance of the selected isolates to one or more antibiotics did not proceed to the molecular identification and characterization of AMR genes. In both cases, the interpretation of the data in terms of the potential effects of gene transfer to other bacteria is impaired. Overall, simultaneous detection of phenotypic and genotypic AMR, which represents the most informative approach, should be considered a standard procedure. However, as shown in Table 1, it was reported in slightly less than half (n = 22) of the analyzed articles.

As for the molecular identity of the detected AMR determinants, there was a clear prevalence of tetracycline (*tet*A, B, K, L, M, O, S, W) and erythromycin (*erm*A, B, C) resistance genes, confirming the results reported in previous studies in foodborne LABs [70,71,72]. The AMR genes were most frequently detected by PCR using known primers (n = 20), while a smaller number of articles (n = 8) retrieved the information by interrogating specific databases (db) with the results of whole genome sequencing (WGS) or shotgun metagenomics. Advanced-omics techniques such as WGS and metagenomics provide more extensive coverage of the bacterial genomes than targeted PCR, but the identification of single genes is strictly dependent on their representation within the available AMR databases. Several different databases collecting AMR gene sequences are presently available (e.g., ARGminer, CARD, MEGARes, NDARO, ResFinder, and SARG), and their updated description was recently reported by [73]. They all represent precious tools for monitoring and tracking AMR spread in bacterial communities, but they differ in the number and type of genes and resistance determinants included. For this reason, it is extremely important to understand the differences among distinct databases to select the best option for a specific purpose. According to [73], CARD and NDARO represent the most suitable tools for AMR annotation, with the NDARO db mostly including acquired resistance genes, while the CARD db comprises a similar number of genes but deriving from a higher number of microbial genera. These features made it especially suitable for AMR mutation screening in a broad range of study settings [73]. However, it should be considered that the CARD and ResFinder dbs which are frequently employed in AMR studies, are mostly based on data from pathogenic bacteria, and the genes from non-pathogenic species are often not included or under-represented [44]. It is therefore advisable to search for AMR genes in more than one database ([44] recommends the KEGG db), especially when retrieving no hits. This aspect points out the crucial importance of creating specific databases to collect sequence data from non-pathogenic AMR bacterial strains. Overall, there is an urgent need for standardization of pipelines and databases as well as phenotypic predictions based on genomic data [74].

**Figure 2:**
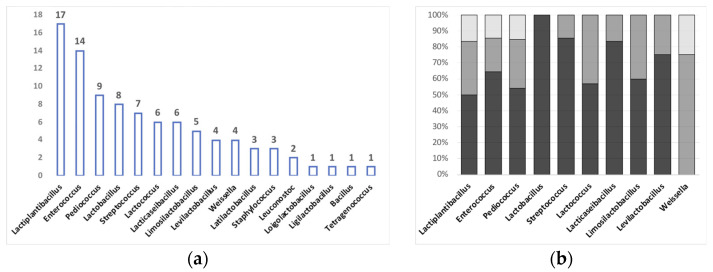
Taxonomical identity of AMR bacteria and their distribution in the different food matrices. (**a**) Number of studies identifying each of the indicated genera among AMR bacteria. Two of the analyzed studies are not represented ([26,56]), as they analyzed the food metagenome and did not report the isolation of specific AMR species/strains; (**b**) Percent occurrence of the AMR genera shown in panel (**a**) within the three fermented food categories. Dairy matrices are represented by the black portion of the bars, plant-derived matrices are shown in dark gray; meat or fish matrices are shown in light gray. The graph shows only the genera that were reported in at least 4 studies in panel (**a**).

As for the taxonomical identification, about one-third of the studies limited their analysis to the original number of isolates without proceeding with strain typing. This limitation did not allow us to perform a numerical comparison of their results with those reported in the other studies describing AMR strains. Different isolates could in fact belong to a single strain, therefore hindering the estimation of the actual frequency of AMR strain distribution within a specific product. On the contrary, most of the analyzed studies (n = 36) focused on a single (or very few) specific AMR species/strains that were pre-selected for further characterization among the foodborne isolates, thus impairing full comprehension of the biodiversity associated with AMR within the food matrix. Finally, two of the studies employed metagenomic analysis of the whole microbial community within the food samples, which was not accompanied by the isolation of AMR bacteria. In these latter studies, the identification of the dominant species in each food sample and the corresponding presence of AMR genes were thus performed independently based on sequence analysis. Although highly informative of the fine structure of the microbial community, this approach does not allow for the precise assignment of AMR determinants to specific taxonomical entities. Figure 2 illustrates the most frequent genera that were identified in the studies reporting taxonomical identification of the AMR isolates.

As shown in panel a, *Lactiplantibacillus* was the most represented genus hosting AMR determinants, closely followed by *Enterococcus*. *Pediococcus*, *Lactobacillus*, *Streptococcus*, *Lactococcus,* and *Lacticaseibacillus*, which are commonly found within the fermented food microbiota, were also frequently isolated. Accordingly, *Staphylococcus* and *Latilactobacillus*, which are mostly represented in meat-based foods that were a small minority of the products analyzed in all countries, were identified in only three studies. Further analysis of the occurrence of each AMR genus within the three major food matrices (dairy, meat/seafood, and vegetable sources) confirmed this observation (Figure 2b). Moreover, *Lactobacillus* and *Streptococcus* were almost exclusively isolated from dairy matrices, while *Weissella* was mostly associated with vegetable sources (Figure 2b).

Along with the identification of AMR genes, the characterization of their genetic context is of utmost importance to evaluate the actual risk of horizontal transfer to other bacteria. Identification of mobile elements in the genomic context of AMR genes is indeed a key factor for evaluating the corresponding risk of HGT. Most of the analyzed articles (n = 40) did not verify the transferability of AMR genes, although two of them [21,28] mentioned the presence of transposon sequences or transposases in the genomic context surrounding the detected AMR genes (Table 1). On the contrary, HGT experiments were performed in nine articles, which are described in more detail in Table 2.

### Horizontal Gene Transfer among Foodborne Bacteria

Since HGT represents a key element in the spread of AMR genes among bacteria, we analyzed in depth the nine articles reported in Table 2, which also includes their methodological details.

**Table 2:** Overview of studies analyzing horizontal gene transfer of antibiotic resistance genes.

Donor	Source	AMR Gene(s)	Genomic Context (Chromosome/ Mobile Element/ Plasmid)	Recipient	Donor: Recipient Ratio	Conjugation Method	Frequency	Confirmation of Transconjugants	Other AMR Genes Not Transferred	Reference
*Pediococcus pentosaceus* OBK05	Buttermilk	Trimethoprim (gene not identified)	Plasmid	*Enterococcus faecalis* *Staphylococcus aureus* *Klebsiella* *pneumoniae* *Escherichia coli*	1:1	Filter mating	2 × 10^−4^ 1 × 10^−6^ 2 × 10^−5^ 3 × 10^−4^	Phenotypic (Kirby-Bauer disc diffusion method)	*aph*(3″)-III, *str*A, *van*A, norfloxacin	[29]
				*Enterococcus faecalis* *Staphylococcus aureus* *Klebsiella pneumoniae* *Escherichia coli*	1:1	Food mating (cheese)	2.5 × 10^−4^ 2 × 10^−6^ 3 × 10^−5^ 3.4 × 10^−4^			
*Enterococcus faecalis* f1	Fermented pork	*aad*E (Streptomycin)	not indicated	*Enterococcus faecalis* f8	1:9	Solid agar mating	1.32 × 10^−7^	Molecular (RAPD, Rep-PCR; PCR detection of AMR genes)	None	[45]
*Lactobacillus delbrueckii* subsp. *bulgaricus* R6	Yogurt	*tet*M (Tetracycline)	not indicated	*Listeria monocytogenes* L82	1:1	Filter mating	7.3 × 10^−7^	Phenotypic (*Listeria monocytogenes* biochemical identification kit); Molecular (PCR detection of AMR genes)	*erm*B, *aac*(6′)-*aph*(2″), *ant*(6), *sul*I, *sul*II, *tet*M, *tet*S from 18 strains	[24]
*Lactiplantibacillus* *plantarum* R41		*tet*S (Tetracycline)	not indicated	*Listeria monocytogenes* L82	1:1	Filter mating	2.9 × 10^−6^			
*Enterococcus faecalis* (14 strains) *Enterococcus faecium* (5 strains)	Ready-to-eat dishes	tetM (Tetracycline)	Tn916/Tn1545	*Enterococcus faecalis* JH2-2 (LMG 19456)	not indicated	Filter mating	1.3 × 10^−6^ to 8.7 × 10^−7^	Molecular (genotyping by PCR melting profile; PCR detection of AMR genes)	*aph*(2″)-Ib, *aph*(2″)-Ic, *aph*(2″)-Id, *ant*(4′)-Ia, *tet*K, *tet*W, *erm*C	[64]
		*erm*B (Erithromycin)	not indicated				3.2 × 10^−6^ to 2.4 × 10^−8^			
		aac(6′)-Ie-aph(2″) (Aminoglycosides)	not indicated				1.7 × 10^−6^ to 3.2 × 10^−8^			
		*ant*(6′)-Ia, *tet*L, *tet*O, *erm*A, *erm*B, *msr*C, *mef*AB	not indicated				Various ranges/different combinations			
*Enterococcus faecium* UC7251 (multi-resistant strain)	Fermented dry sausage	*tet*M (Tetracycline)	Tn916/Tn1545	*Enterococcus faecalis* OG1RF *Listeria innocua L7* *Listeria monocytogenes DSM 15675* *Staphylococcus aureus UC7180* *Lacticaseibacillus rhamnosus UC8647*	1:1	Filter mating	6 × 10^−3^ 5.7 × 10^−6^ 8.4 × 10^−4^ 3.8 × 10^−2^ 6.8 × 10^−5^	Molecular (PCR detection of AMR genes	*Tet*L and unknown gene conferring erythromycin resistance on a mobilizable but non-conjugative plasmid lacking the complete conjugation apparatus. No gene transfer observed toward Gram-negative recipient species	[60]
*Limosilactobacillus fermentum* DVM 95.7 and *Limosilactobacillus fermentum* NIFTEM 95.8	Fermented milk	*tet*M, *erm*B	Chromosomal (*tet*M); plasmid (*erm*B)	*Enterococcus faecalis* ATCC 14506, *Escherichia coli* ATCC 11229, *Staphylococcus aureus* NCDC 109	1:10	Filter mating	6.0–6.4 × 10^−6^ with *E. coli*; no transconjugants with other recipients	none	none	[35]
					1:1	Food mating (fermented milk, idli batter, fermented minced chicken, plant model)	2.0 × 10^−1^–3 × 10^−2^ with *E. coli* and *E. faecalis* in minced chicken and in plant model; no transconjugants with *S. aureus*			
*Pediococcus pentosaceus* (15 isolates)	Fermented dairy and meat	*erm*B, *msr*C	Plasmid	*Enterococcus faecalis* JH2-2	50:1	Filter mating	1.0 × 10^−6^ (only for 1 strain, only ermB transferred)	Molecular (genotyping by RAPD-PCR; detection of AMR genes by PCR and Southern hybridization)	none	[32]
*Ligilactobacillus salivarius* (CHS-1E and CH7-1E strains) and *Limosilactobacillus reuteri* (CH2-2 strain)	Fermented chicken sausage	*erm*B, *tet*W, *tet*M, *tet*L	Plasmid	*Enterococcus faecalis* JH2-2	10:1, 25:1 and 50:1	Filter mating	2 × 10^−3^–1 × 10^−4^ (only 50:1 ratio)	Molecular (genotyping by RAPD-PCR;	none	[33]
				*Enterococcus faecalis* JH2-2	1:10	In vivo mating (Wistar rats)	not indicated (although transconjugants were recovered)	detection of AMR genes by Southern hybridization and/or PCR)		
				*Enterococcus faecalis* JH2-2, *Listeria monocytogenes Scott A*, *Micrococcus luteus*, *Yersinia enterocolitica*, *Bacillus cereus* F4453, *Staphylococcus aureus*, pathogenic *E. coli* MTCC118	1:10	Food mating (chicken sausage, fermented milk, idli batter)	1.9 × 10^−6^–2.9 × 10^−7^ (*Listeria*), 0.8 × 10^−6^–0.8 × 10^−9^ (*Yersinia*)			
*Staphylococcus saprophyticus* KM1053	Jeotgal (High-salt fermented seafood)	*tet*K	Plasmid	*Enterococcus faecalis OG1RF*	1:10	Broth mating	5.8 × 10^−3^	Molecular (16S rRNA gene sequence analysis; PCR detection of AMR genes)	none	[43]
				*Staphylococcus equorum KM1031*			9.5 × 10^2^			
				*Staphylococcus equorum KS1039*			not transferred			
				*Staphylococcus aureus USA300 LAC*			not transferred			

All nine articles summarized in Table 2 employed in vitro approaches to test conjugal transfer of AMR genes. Among them, filter mating resulted in the predominant conjugal method, followed by food mating, but also broth mating and solid agar mating were employed. Even though mating assays within a food matrix can better mimic the natural environment, all in vitro approaches can often be misleading as they do not reflect a real-life condition, while conjugal transfer through in vivo mating is more reliable [75], especially when aiming to evaluate the risk of AMR transmission to human pathogens. However, this latter technique is less frequently adopted, and it was reported in only one of the works analyzed in the present review [33]. In particular, the cited article reports both in vivo (rodents) and in vitro (food mating) approaches using the same recipient strain (*E. faecalis* JH2-2). However, the results of in vivo mating were described only in terms of the positive recovery of transconjugants without providing the corresponding transfer frequencies, while in vitro conjugation resulted in a transfer frequency of 10^−6^. We cannot therefore compare the results obtained with the two experimental approaches. As for the transfer frequencies reported in the nine articles, they were highly variable among the different studies, spanning from 10^−1^ to 10^−9^. Overall, low frequencies were observed in most cases, ranging from 10^−6^ to 10^−7^, but some articles reported higher transfer frequencies (10^−1^–10^−2^), especially in food mating systems [35]. Such an ample range of transfer frequencies cannot be attributed only to differences in the recipient strains and/or to the association of the AMR gene(s) with mobile genomic elements but also appears to involve other methodological aspects that were not standardized in the studies, such as the donor/recipient ratios. Although 1:1 was the most frequent ratio employed, some articles reported using ten times the amount of recipient (1:10 ratio), while in some others a much higher proportion of donor was employed with respect to the recipient (e.g., 10:1, 50:1, 25:1 reported by [32,33]). Overall, the crucial factors affecting transfer frequencies appear to reside in the employed methodologies as well as in the taxonomical identity of the recipient strains, which were mainly represented by the commonly used *E. faecalis* strain JH2-2. However, several other different pathogens were used as recipients, for example, *S. aureus*, *K. pneumoniae*, *E. coli*, and *L. monocytogenes*. In one case, a LAB strain was used as a recipient (*Lacticaseibacillus rhamnosus* strain UC8647, in [60]), but all other studies employed Gram-positive (*Bacillus*, *Enterococcus*, *Listeria*, *Micrococcus*, *Staphylococcus*) or Gram-negative (*Escherichia*, *Klebsiella*, *Yersinia*), human pathogens, or opportunistic pathogens. In all cases, the donor strains were represented by foodborne LAB, whose isolation from the different food matrices is described in Table 1.

As shown in Table 2, meat and dairy were the most common sources of donor AMR strains, mainly represented by species belonging to the former *Lactobacillus* genus and by *Enterococcus faecalis* and *faecium* species, followed by *Pediococcus pentosaceus* and *Staphylococcus saprophyticus*. Concerning the choice of specific resistance determinants, transfer of *tet* and *erm* genes was prevalent, in particular *tet*M and *erm*B, respectively. This could be related to the frequent association of such genes with the conjugative transposon Tn916, conferring high transmission capacity [72,76]. Indeed, when tested in filter mating experiments, other *tet* and *erm* genes carried by the same isolates but not associated with transposons or other conjugative elements did not show any transfer to the recipient strains [60,64].

Aminoglycoside and streptomycin resistance genes were shown to be capable of HGT in *E. faecalis* strains in two articles performing in vitro mating on filter or solid agar [45,64]. Horizontal transfer of trimethoprim resistance from the *P. pentosaceus* strain OBK05 to four different Gram-positive pathogens was also shown by mating assays within a cheese matrix and was hypothesized to be mediated by a plasmid. However, the corresponding resistance gene was not identified but was only supposed to be plasmid-encoded since plasmid curing impaired the acquisition of the resistant phenotype by the recipient strains [29]. Noteworthy, conflicting results concerning transferability were obtained for some of the *tet* and *erm* genes: for example, the *erm*B, *tet*M and *tet*S genes were successfully transferred from *L. delbrueckii* ssp. *bulgaricus* strain R6 and *Lactiplantibacillus plantarum* strain R41 to *Listeria monocytogenes*, but when using 18 other donor strains no transfer of the same genes was observed toward the same recipient [24]; *tet*K was successfully transferred from the *Staphylococcus saprophyticus* strain KM1053 to two out of four *Enterococcus*/*Staphylococcus* recipients [43], while it was not mobilizable from *E. faecalis* and *E. faecium* donors to the recipient *E. faecalis* strain JH2-2 [64]; finally, the macrolide/streptogramin B resistance gene *msr*C was reported to be mobilized from *E. faecalis* and *E. faecium* donors to the *E. faecalis* strain JH2-2 recipient by filter mating [64], but transfer experiments from *P. pentosaceus* isolates to the same *E. faecalis* JH2-2 recipient within food matrices were unsuccessful [32]. Aside from the experimental details, such discrepancies could also derive from the specific genomic context of the AMR genes, which is a crucial determinant of the transmission capacity. Among the papers analyzed and collected in Table 2, information regarding the genomic context of the identified AMR genes was not available in three articles [24,45,64], while in most articles it was indicated, especially for *tet* and *erm* genes that were shown to be associated with plasmids or transposons (Tn916) and to be capable of horizontal transfer [32,33,35,43,60,64]. One exception was represented by a plasmid borne *tet*L, harbored by the *E. faecium* strain UC7251, which was not transferred to Gram-negative recipients, probably due to the non-conjugative nature of the plasmid, which was lacking a complete conjugation apparatus [60].

Another methodological aspect that should be better standardized concerns the techniques used to confirm the transconjugants. Indeed, phenotypic AMR as the only confirmation method is often unreliable, and, as suggested by other authors, the combination of molecular analysis with phenotypic tests is recommended [71]. Among the molecular approaches, detection of AMR genes by means of PCR or Southern hybridization proves the effective transfer of such genes to the recipient strains, and these methods were adopted in most articles reported in Table 2, while two of them applied only phenotypic tests [29] or no confirmation [35]. Moreover, in addition to the molecular detection of the transferred AMR genes, the genotyping of the transconjugants provides complete proof of a successful HGT event. Four of the analyzed studies performed genotyping of transconjugants coupled to the molecular detection of AMR genes [32,33,43,64].

In conclusion, our attempt to perform a comparison of the relevant literature published in the past five years and reporting the isolation and molecular characterization of foodborne AMR bacteria points to the methodological heterogeneity as a major weakness preventing a causal relationship between the presence of AMR determinants within the microbial reservoir associated with fermented foods and their transmission to human pathogens. This important limitation affects, in turn, the possibility of assessing the actual risk posed by the foodborne environmental reservoir for human health, which represents the basis for developing policies aimed at preventing the uncontrolled spread of AMR.

## 5. Global/Regional Policies to Tackle AMR Spread

Effective action to tackle the spread of AMR requires constant monitoring. Within the EU, the European Food Safety Authority (EFSA) coordinates annual monitoring of AMR in pathogenic bacteria from food and livestock and provides scientific advice on risk assessment models and possible strategies to counteract the environmental increase of AMR bacteria [77]. The use of antibiotics as feed additives in animal farming has been banned in all EU countries since January 2006, and guidelines for the proper use of antibiotics were more recently published by EU authorities [78]. Similarly, the World Health Organization (WHO) imposed a total restriction on the use of antibiotics as growth promoters in 2017 [79]. Among the extra-EU countries, China, where a large amount of antibiotics in livestock husbandry has traditionally been employed, recently adopted a national plan for their reduction in animal feed [80].

At a global level, the AMR Inter-Departmental Working Group established by the Food and Agricultural Organization (FAO) in 2015 has recently delivered an updated action plan for the years 2021–2025 with several important objectives, including strengthening surveillance and research on AMR spread, promoting responsible use of antimicrobials, and enabling good practices. The document highlights the utmost importance of closing the gaps in current knowledge on the mechanisms of AMR spread and the urgency of addressing this growing global threat in all countries through a coordinated, multisectoral, One Health approach in the context of the UN 2030 Agenda for Sustainable Development [81].

Within this context, the International Union of Microbiological Societies (IUMS) recently launched a call to action directed to all microbiological societies worldwide, highlighting the need to develop science-based policies aimed at preventing the spread of AMR while also preserving the microbial ecosystem and its biodiversity by developing sustainable solutions to control infectious agents [82]. The overuse and misuse of antibiotics is indeed one of the human actions that had a strong impact on microorganisms, but it should not be forgotten that microbial biodiversity within environmental niches also contributes essential functions that support life on Earth, falling within the domain of One Health issues.

From a policy viewpoint, tackling AMR as a One Health issue represents an extremely important step to raise stakeholders’ awareness and elicit a global response from the food and agricultural sectors in the fight against AMR. Moreover, the active involvement of inter-governmental organizations such as FAO, WHO, and OIE, which are supported worldwide, ensures the alignment of strategic and financial efforts to sustain science-based and international guidance in the fight against AMR spread.

From the scientific viewpoint, further investigations are needed to close the knowledge gaps, but their usefulness relies on the possibility to compare the results of studies performed in different settings on different foods and microbial environments, which requires a high degree of methodological standardization, pointing to the urgent need for aligning standard operating procedures (SOPs) and pipelines. Efforts in this direction have been in place at the European and international levels since the first decade of this millennium, resulting in the development of analytical protocols for the quantification of AMR in food-grade bacteria. Factors affecting the results of phenotypic AMR assays, such as the composition of the growth medium, the inoculum size, and the time and temperature of incubation, have been identified, and the experimental protocols for determining the minimum inhibitory concentration (MIC) for antibiotics are presently applied with fully standardized procedures. Reference cut-off values to discriminate resistant and susceptible strains have also been uniquely defined in a document published and periodically updated by EFSA [83]. Highlighting the importance of monitoring the presence of AMR in strains deliberately used as feed additives or as production organisms, the EFSA also recommended determining the whole genome sequence of AMR strains in addition to the phenotypic resistant profile for reliable identification of AMR genes [84]. These standardized, shared, and internationally accepted SOPs to assess the phenotypic AMR profile of non-pathogenic foodborne bacteria, coupled with the application of WGS to detect the presence of the corresponding AMR genes, should therefore be more generally applied to achieve comparable results. To date, genomic data of food-associated bacteria is still scarce, hindering the possibility of performing in silico analysis that would be extremely helpful to achieve a more accurate identification of AMR traits, including their genomic context (chromosomal, plasmid-encoded, or associated with mobile elements), which is strongly predictive of their potential horizontal transferability. In line with the increasing importance of delivering high-quality scientific results that could be shared through open-access databases and analyzed by advanced computation, the last two European research frameworks (Horizon 2020 and Horizon Europe) supported the development and shared use of research infrastructures (RIs) with dedicated funding. RIs are facilities, resources, and services used by the research communities to conduct research and foster innovation in their fields. The definition of RIs in Article 2 (6) of the EU Regulation No. 1291/2013 (Establishing Horizon 2020—the Framework Programme for Research and Innovation 2014–2020) also includes knowledge-based resources such as collections, archives, and scientific data among the tools that are essential to achieving excellence in research and innovation (https://www.esfri.eu/glossary). The initial and crucial efforts addressed by the scientific community toward infrastructural development were therefore aimed at standardizing SOPs and pipelines to produce datasets that could meet the FAIR (i.e., Findable, Accessible, Interoperable, and Reusable) principles. These standardization efforts have impacted microbiological research as well [1,85,86]. Future investigations monitoring AMR spread within environmental reservoirs and its potential transmission to human pathogens should therefore align with these efforts by including a minimum set of standardized procedures in their study design so that the results of individual studies can be compared and a more accurate picture of the actual frequencies of horizontal transfer can emerge.

## Figures and Tables

**Table 1:** List of experimental studies analyzed in the present review reporting the isolation or characterization of AMR bacteria associated with fermented foods. The studies are listed according to the continent of origin.

AMR Species Identified	Food Source	Food Origin	Characterization of Bacterial Isolates	Phenotypic Resistance	Detected AMR Genes	Detection Method of AMR Gene(s)	Reference
**Asia**							
*Lactococcus lactis*, *Lactobacillus gallinarum*, *Lacticaseibacillus casei*, *Lacticaseibacillus paracasei*, *Lactiplantibacillus plantarum*, *Streptococcus thermophilus*	Fermented dairy products (Mozzarella, Traditional milk tofu, Cheddar, Cream, Mimolette Wedge, Emmentaler, Brie, Feta)	China	16S-DNA-based metagenomics (cheese metagenome); 16S rRNA gene sequencing (isolates)	Streptomycin, sulfamethoxazole, clindamycin, tetracycline, penicillin, norfloxacin, ciprofloxacin	*aad*E, *str*A, *str*B, *sul*1, *sul*2	qPCR	[21]
*Streptococcus thermophilus*, *Lactobacillus bulgaricus*, *Lactobacillus acidophilus*, *Lactiplantibacillus plantarum*	Fermented dairy products (Yogurt)	China	16S rRNA gene sequencing	Streptomycin, tetracycline, vancomycin	not determined	not determined	[22]
*Lactococcus*, *Leuconostoc mesenteroides*, *Weissella cibaria/soli/confusa, Enterococcus gallinarum/durans/hirae*, *Pediococcus pentosaceus*, *Bacillus coagulans*, *Lactococcus garvieae/lactis*	Fermented vegetable products (Broccoli, Cherry, Ginger, White radish, White-fleshed pitaya juices)	China	16S rRNA gene sequencing	Ampicillin, penicillin, amoxycillin, orfloxacine, levoflacin, gentamycin, streptomycin, amikacin, erythromycin	not determined	not determined	[23]
*Lactobacillus delbrueckii* subsp. *bulgaricus*, *Lactiplantibacillus plantarum*, *Streptococcus thermophilus*	Fermented dairy products (Yogurt)	China	16S rRNA gene sequencing; PFGE	Erythromycin, gentamycin, streptomycin, sulfamethoxazole, tetracycline	*erm*B, *aac*(6′)-*aph*(2″), *ant*(6), *sul*I and *sul*II, *tet*M, *tet*S	PCR	* [24]
*Lactiplantibacillus plantarum*, *Lactiplantibacillus fermentum*	Fermented vegetable products (Yucha)	China	Shotgun metagenomics; 16S rRNA gene sequencing of isolates	not determined	AMR genes (not specified)	Shotgun metagenomics, Comprehensive Antibiotic Resistance Database (CARD)	[25]
Food metagenome	Fermented vegetable products	China	Shotgun metagenomics	not determined	Multidrug resistance genes	Shotgun metagenomics, SARG database	[26]
*Staphylococcus carnosus*, *Lactiplantibacillus plantarum*, *Latilactobacillus sakei*, *Weissella cibaria*, *Weissella confusa*	Fermented meat products	China	RAPD; 16S rRNA gene sequencing	Streptomycin, vancomycin, erythromycin, roxithromycin, lincomycin, kanamycin	*tet*M, *ere*A, *str*A, *str*B	PCR	[27]
*Streptococcus thermophilus*	Fermented dairy products (Koumiss, Kurut, Fermented cow milk, Fermented goat milk, Qula)	China, Mongolia	WGS	Chloramphenicol	*van*H, *van*U, *cat*B8	WGS, Comprehensive Antibiotic Resistance Database (CARD); droplet digital PCR	[28]
*Pediococcus pentosaceus*	Fermented dairy products	India	16S rRNA gene sequencing	Kanamycin, streptomycin, vancomycin, ciprofloxacin, norfloxacin (chromosomal); trimethoprim (plasmid)	*aph* (3″)-III, *str*A, *van*A, *gyr*A	PCR	* [29]
*Enterococcus faecalis*	Fermented fish products (Tungtap)	India	16S rRNA gene sequencing	Kanamycin, gentamycin, streptomycin, ciprofloxacin, vancomycin, penicilin G, ampicillin, tetracyclin, rifampycin, chloraphenicol, polymyxin b	*van*A, *van*B	PCR	[30]
*Levilactobacillus brevis*, *Enterococcus durans*, *Enterococcus lactis*, *Enterococcus faecium*, *Leuconostoc lactis*	Fermented vegetable products (Coconut palm nectar)	India	16S rRNA gene sequencing	Ampicillin, vancomycin, gentamicin, kanamycin, chloramphenicol, erythromycin, clindamycin	not determined	not determined	[31]
*Pediococcus pentosaceus*	Fermented foods (Idli, Dosa batter, Dahi, Fermented dry sausage)	India	not indicated	Azitromycin, clarithromycin, clyndamicin, lyncomincin, piramicin, pristinamycin, streptograminB	*erm*B, *msr*C	PCR, Southern blot	* [32]
*Ligilactobacillus salivarius*	Fermented poultry products	India	16S rRNA gene sequencing	Erythromycin, tetraciclyne	*erm*B, *tet*W, *tet*M, *tet*L	PCR, Southern blot	* [33]
*Pediococcus pentosaceus*, *Pediococcus acidilactici*	Fermented food products	India	species-specific PCR	Vancomycin, nalidixic acid	not determined	not determined	[34]
*Limosilactobacillus fermentum*	Fermented dairy products	India, China	Phenotipic tests	Ampicillin, ciprofloxacin, vancomycin, streptomycin, trimethoprim	*tet*M, *erm*B	PCR	* [35]
*Lactobacillus acidophilus*, *Limosilactobacillus reuteri*	Fermented vegetable and dairy products	Indonesia	not indicated	Streptomycin, kanamycin, amikacin, meropenem	not determined	not determined	[36]
*Enterococcus faecalis*, *Enterococcus faecium*	Fermented dairy products	Iran	species-specific PCR (ddl gene)	Quinupristin/dalfopristin, penicillin G, ampicillin, rifampicin, doxycycline, ciprofloxacin	not determined	not determined	[37]
*Enterococcus faecalis*, *Enterococcus faecium*	Fermented dairy products	Iran	16S rRNA gene sequencing	Rifampicin, quinupristin, dalfopristin	*ace*, *gel*E, *asa1*	PCR	[38]
*Lactobacillus harbinensis,* *Lacticaseibacillus paracasei*, *Lactiplantibacillus plantarum*	Fermented dairy products (Kefir)	Malaysia	16S rRNA gene sequencing	Ampicillin, penicillin, tetracycline	not determined	not determined	[39]
*Lactiplantibacillus plantarum*, *Lactobacillus helveticus*	Fermented dairy products (Yogurt)	Pakistan	16S rRNA gene sequencing	Ampicillin, trimethoprim, vancomycin, nitrofurantoin	not determined	not determined	[40]
*Lactiplantibacillus plantarum*	Fermented vegetable products	Republic of Korea	16S rRNA sequencing	Chloramphenicol, tetracycline	not determined	not determined	[41]
*Lactiplantibacillus plantarum*	Fermented vegetable products	Republic of Korea	WGS	Ampicillin	*erm*B	WGS, Comprehensive Antibiotic Resistance Database (CARD)	[42]
*Staphylococcus saprophyticus*	Fermented seafood products (Jeotgal)	Republic of Korea	16S rRNA gene sequencing	Penicillin G, tetracycline	*tet*K	PCR	* [43]
*Lactiplantibacillus plantarum*	Fermented meat products (Nham)	Thailand	WGS	Chloramphenicol, kanamycin	*cat*	PCR	[44]
*Enterococcus faecium*, *Enterococcus faecalis*	Fermented meat products	Thailand	not indicated	Streptomycin	*aad*E	PCR	* [45]
*Levilactobacillus brevis*, *Lactiplantibacillus plantarum*, *Lacticaseibacillus paracasei*, *Loigolactobacillus coryniformis*, *Lacticaseibacillus rhamnosus*, *Lactobacillus helveticus*	Fermented dairy products (Tulum)	Turkey	16S rRNA gene sequencing	Ampicillin, chloraphenicol, erytromycin, gentamycin, kanamycin, penicillin, streptomycin, tetracycline, vancomycin	not determined	not determined	[46]
*Enterococcus faecalis*, *Enterococcus faecium*, *Enterococcus durans*, *Enterococcus gallinarum*	Fermented dairy products	Turkey	species-specific PCR	Streptomycin, gentamicin	*aph*(3′)*-*IIIa, *ant*(4′)-Ia, *ant*(6′)-Ia, *aph*(2″)*-*Ic	PCR	[47]
**Africa**							
*Limosilactobacillus fermentum*, *Weissella confusa*, *Lactiplantibacillus plantarum*, *Pediococcus pentosaceus*, *Pediococcus acidilactici*	Fermented cereal products (Mawé)	Benin	16S rRNA gene sequencing	Chloramphenicol, erythromycin, tetracycline, ampicillin, streptomycin, kanamicin	*aph*(3)I, *aph(*3)III, *tet*S, *tet*M, *tet*L, *str*A, *str*B, *aad*A, *aad*E, *str*A, *str*B, *aad*A, *aad*E	PCR	[48]
*Enterococcus faecium*, *Enterococcus faecalis*	Fermented dairy products (Yogurt, Cheese, Milk)	Ethiopia	species-specific PCR	Erythromycin, tetracycline, ampicillin, oxacillin, ciprofloxacin, azithromycin, vancomycin	*van*C1, *van*C2/C3, *erm*B, *erm*C	PCR	[49]
*Enterococcus fecalis*	Fermented dairy products (Testouri cheese, Rigouta, Yogurt, Leben, Rayeb)	Tunisia	MALDI TOF, species-specific PCR, WGS	Gentamicin, erythromycin	Tetracycline resistance determinants (not specified)	WGS	[50]
*Lactiplantibacillus plantarum*, *Limosilactobacillus fermentum*	Fermented cereal products (Mahewu)	Zimbawe	WGS	Streptomycin, norfloxacin, erythromycin, chloramphenico, tetracycline	*tet*B(48), *tet*A(48), *efr*A, *efr*B, *dfr*E, *dfr*F, *dfr*I, *lmr*B, *lmr*D, *mdt*G, *van*RM, *van*RF, *van*HF, *pat*A, *pat*B, *qac*H, *eme*A, *mpr*F, *bac*A, *tae*A, *fus*F	WGS, Comprehensive Antibiotic Resistance Database (CARD)	[51]
**South America**							
*Enterococcus faecium*, *Lactococcus lactis*, *Lacticaseibacillus rhamnosus*, *Weissella cibaria*	Fermented cereal products (Chia sourdough)	Argentina	16S rRNA gene sequencing	Clindamycin, erythromycin, gentamycin, vancomycin	not determined	not determined	[52]
*Levilactobacillus brevis*, *Lacticaseibacillus paracasei*, *Lactococcus lactis*, *Lacticaseibacillus rhamnosus*, *Lactobacillus pentosaceus*, *Lactilactobacillus curvatus*, *Lactiplantibacillus paraplantarum*, *Pediococcus acidilactici*	Fermented dairy products	Brasil	MALDI-TOF	Gentamycin, ciprofloxacin, vancomycin, streptomycin	not determined	not determined	[53]
*Enterococcus hirae*, *Pediococcus pentosaceus*	Fermented dairy products	Brazil	RAPD; 16S rRNA gene sequencing	Ampicillin, penicillin G, oxacillin, clindamycin, erythromycin, imipenem, rifampicin, chloramphenicol, tetracycline, trimethoprim/sulfamethoxazole vancomycin	*erm*A, *erm*B, *erm*C, *bcr*B, *tet*O, *vat*E	PCR	[54]
*Streptococcus thermophilus*	Fermented dairy products (Buffalo mozzarella cheese)	Brazil	16S rRNA gene sequencing	Oxacillin	not determined	not determined	[55]
Food metagenome of different cheese samples: *Lactococcus lactis*, *Streptococcus thermophilus-salivarius*, *Streptococcus equinus-lutetiensis-infantarius* complex (dominant species); *Lacticaseibacillus casei*, *Lactobacillus delbrueckii*, *Lactiplantibacillus plantarum*, *Lactococcus piscium*, *Leuconostoc mesenteroides*, *Streptococcus* spp., *Lactococcus* spp. (less abundant species)	Fermented dairy products	Brazil	16S-DNA-based metagenomics; Shotgun metagenomics	not determined	30 AMR genes identified, belonging to nine different classes of antibiotics (*tet*K, *tet*S most prevalent)	Shotgun metagenomics, Comprehensive Antibiotic Resistance Database (CARD)	[56]
**Europe**							
*Enterococcus faecalis*, *Enterococcus durans*, *Enterococcus faecium*	Fermented dairy products (Ragusano, Pecorino)	Italy	MALDI-TOF, species-specific multiplex PCR	Tetracycline, erythromycin, streptomycin, gentamycin, ampicillin, rifampicin, penicillin G, sulphametoxazol, chloramphenicol, vancomycin, kanamycin	not determined	not determined	[57]
*Lactiplantibacillus plantarum*	Fermented foods (Table olives, Pickled cabbage, Sourdough, Raw milk cheese)	Italy	multilocus sequence typing (MLST)	Vancomycin, ciprofloxacin; streptomycin, kanamycin; gentamicin, penicillin	not determined	not determined	[58]
*Streptococcus macedonicus*	Fermented dairy products	Italy	WGS	Kanamycin	not determined	not determined	[59]
*Enterococcus faecium*	Fermented dry sausage	Italy	species-specific PCR	Ampicillin, streptomycin, kanamycin, erythromycin, clindamycin, tylosin, tetracycline	*erm*B, *tet*M, *tet*L, *aph*3-IIIa, *sat*A, *ant*(6)-Ia, *aad*E	PCR	* [60]
*Latilactobacillus sakei*, *Lactiplantibacillus plantarum*, *Staphylococcus xylosus*, *Staphylococcus equorum*, *Staphylococcus saprophyticus*	Fermented meat products (Italian Piacentino Salami DOP; Fermented pork and llama meat products)	Italy, ** Argentina	RAPD; 16S RNA gene sequencing	Tetracycline, erythromycin	*tet*M, *tet*K, *tet*W, *tet*S, *tet*L, *erm*A, *erm*B, *erm*C	PCR	[61]
*Lactococcus lactis*	Raw milk	Lithuania	species-specific PCR	Clindamycin, streptomycin, gentamycin, tetracycline erytromycin, ampicillin, vancomycin, kanamycin	not determined	not determined	[62]
*Enterococcus faecium*, *Enterococcus faecalis*	Fermented dairy products	Poland	species-specific PCR	Streptomycin, erythromycin, tetracycline, rifampicin, tigecycline, vancomycin, linezolid	*ant*(6′)-Ia, *aac*(6′)-Ie-*aph*(2″)-Ia, *aph*(3″)-IIIa; *tet*M, *tet*L; *erm*A, *erm*B	PCR	* [63]
*Enterococcus faecium*, *Enterococcus faecalis*	Ready-to-eat foods	Poland	RAPD	Erythromycin, gentamicin, streptomycin, tetracycline, tigecycline, fosfomycin, ciprofloxacin, levofloxacin, norfloxacin, rifampicin, linezolid, quinupristin/dalfopristin	*aac*(6′)-Ie-*aph*(2″)-Ia, *aph*(2″)-Ib, *aph*(2″)-Ic, *aph*(2″)-Id, *ant*(4′)-Ia, *ant*(6′)-Ia; *tet*M, *tet*L, *tet*K, *tet*O, *tet*W, *erm*A, *erm*B, *erm*C, *msr*C, *mef*AB	PCR	[64]
*Enterococcus faecium*, *Levilactobacillus brevis*, *Limosilactobacillus fermentum*, *Lacticaseibacillus paracasei*, *Lactiplactibacillus paraplantarum*, *Lactococcus lactis*, *Leuconostoc mesenteroides*, *Leuconostoc pseudomesenteroides*	Fermented dairy products	Slovakia	WGS	not determined	*aac*(6′)-li, *msr*C, *efm*A, *lmr*CD	WGS, Comprehensive Antibiotic Resistance Database (CARD)	[65]
*Tetragenococcus koreensis*, *Tetragenococcus halophilus*	Fermented dairy products	Spain	16S rRNA gene sequencing	Erythromycin, clindamycin	not determined	not determined	[66]
*Enterococcus faecium*, *Enterococcus mundtii*, *Enterococcus hirae*	Fermented legumes	Sweden	MALDI-TOF	Ampicillin, trimethoprim, vancomycin, nitrofurantoin	not determined	not determined	[67]
*Pediococcus pentosaceus*	Fermented dairy and meat products	Switzerland	WGS, MALDI-TOF	Ampicillin, gentamycin, erytromycin, clindamycin, tetracycline, chloramphenico, streptomycin, vancomicin, kanamycin	not determined	WGS AMRFin-derPlus, CARD, ARG-ANNOT, and Resfinder databases	[68]
*Pediococcus acidilactici*	Fermented dairy products (Gruyere, Emmental, Tete de Moine, Sbrinz, Tilsit)	Switzerland	MALDI-TOF	Clindamycin, tetracycline, streptomycin, penicillin, chloramphenicol, kanamycin, vancomycin, gentamycin, erythromycin	not determined	not determined	[69]

* Described in detail in Table 2; ** This work analyzed products from both Italy and Argentina but was included exclusively in the group of Europe as continent of origin to avoid duplication.

## Data Availability

Not applicable.
